# Expression and Ion Transport Activity of Rice *OsHKT1;1* Variants

**DOI:** 10.3390/plants9010016

**Published:** 2019-12-21

**Authors:** Shahin Imran, Tomoaki Horie, Maki Katsuhara

**Affiliations:** 1 Institute of Plant Science and Resources, Okayama University, 2-20-1, Chuo, Kurashiki 710-0046, Japan; ptj87a5q@s.okayama-u.ac.jp; 2 Division of Applied Biology, Faculty of the Textile Science and Technology, Shinshu University, 3-15-1, Tokida, Ueda 386-8567, Japan

**Keywords:** *HKT*, rice, salt tolerance, Na^+^ transport, K^+^ transport, mRNA variants

## Abstract

*OsHKT1;1* in rice, belongs to the high-affinity K^+^ Transporter family, has been found to be involved in salt tolerance. *OsHKT1;1* in *japonica* rice (Nipponbare) produces mRNA variants, but their functions remain elusive. In salt tolerant rice, Pokkali, eight *OsHKT1;1* variants (V1-V8) were identified in addition to the full-length *OsHKT1;1* (FL) cDNA. Absolute quantification by qPCR revealed that accumulation of *OsHKT1;1-FL* mRNA is minor in contrast to that of *OsHKT1;1-V1*, *-V2*, *-V4*, and *-V7* mRNAs, all of which are predominant in shoots, while only *V1* and *V7* mRNAs are predominant in roots. Two electrode voltage clamp (TEVC) experiments using *Xenopus laevis* oocytes revealed that oocytes-expressing OsHKT1;1-FL from Pokkali exhibited inward-rectified currents in the presence of 96 mM Na^+^ as reported previously. Further TEVC analyses indicated that six of eight OsHKT1;1 variants elicited currents in a Na^+^ or a K^+^ bath solution. OsHKT1;1-V6 exhibited a similar inward rectification to the FL protein. Contrastingly, however, the rests mediated bidirectional currents in both Na^+^ and K^+^ bath solutions. These data suggest possibilities that novel mechanisms regulating the transport activity of OsHKT1;1 might exist, and that OsHKT1;1 variants might also carry out distinct physiological roles either independently or in combination with OsHKT1;1-FL.

## 1. Introduction

Salinity affects plant growth and development and causes low crop productivity [1]. Salinity can give rise to osmotic stress, nutritional imbalance, and ionic toxicity in the plant [2,3]. Among major cereal crops, rice (*Oryza sativa*) is the most sensitive to salinity stress [2,4].

The high-affinity K^+^ transporter (*HKT*) gene family has been found to be relevant to salt stress tolerance in plants [5,6]. The first *HKT* gene in plants, *TaHKT2;1* from bread wheat *Triticum aestivum*, was reported to be a Na^+^-K^+^ co-transporter [7]. HKTs can be classified into at least two subfamilies [5,8]. Subfamily 1 HKTs (HKT1s) are found in monocotyledonous and dicotyledonous species, and they are Na^+^ selective transporters. So far, only monocotyledonous species contain the subfamily 2 HKTs (HKT2s) that are selective for both Na^+^ and K^+^ [9]. Interestingly, both HKT1 and HKT2 proteins were suggested to exhibit features of a Na^+^ channel when the Na^+^ concentration at extracellular spaces are high [7,10,11]. In fact, AtHKT1;1 in *Arabidopsis*, belongs to the subfamily 1, has been demonstrated to show a Na^+^ channel activity by comparative patch clamping analyses using wild-type and *athkt1;1* loss-of-function mutants [12]. In a *japonica* cultivar of rice, seven functional *OsHKT* genes have been identified [13]. Four of them were grouped in the *HKT1* gene subfamily and those products, OsHKT1;1, OsHKT1;3, OsHKT1;4 and OsHKT1;5, were shown to be Na^+^ selective by the two-electrode voltage clamp experiment using *Xenopus laevis* oocytes [14,15,16,17].

A QTL (Quantitative Trait Locus) analysis using a salt tolerance *indica* landrace, Nona Bokra, and a *japonica* cultivar, Koshihikari, has led to the identification of the *shoot K^+^ content 1* (*SKC1*) locus, which regulates accumulation of higher K^+^ and lower Na^+^ in shoots under salt stress [14]. The *OsHKT1;5* gene encoding a plasma membrane-localized Na^+^ transporter was suggested to be a causal gene for the QTL [14]. The SKC1-dependent phenotype in the accumulation of K^+^ and Na^+^ was similar to the mechanism found in the *athkt1;1* mutant of *Arabidopsis* [18,19], which supports a hypothetical model that HKT1-mediated Na^+^ unloading at the xylem indirectly couples with K^+^ loading, which eventually accelerates lower Na^+^ but higher K^+^ accumulation in shoots [5]. More recently, OsHKT1;5 was demonstrated to have a critical role in reducing Na^+^ contents of young leaves of rice under salt stress via Na^+^ unloading at the xylem of roots and leaf sheaths and the phloem of the basal node [20].

Characterization of a *Tos17* mutant rice line, which harbors an insertion of a transposon in the *OsHKT1;1* gene has revealed that OsHKT1;1 plays an important role in reducing Na^+^ accumulation in the shoot to cope with salt stress [21]. The *Tos17*-insertion in *OsHKT1;1* was found to cause a reduction in Na^+^ contents in the phloem sap but an increase in the xylem sap in response to salt stress, similar to *athkt1;1* mutants [18,21]. In addition, the *OsHKT1;1* gene has been strongly suggested to be a determinant of salt tolerance of rice by two independent genetic studies [22,23]. In particular, Campbell et al. [23] indicated that an OsHKT1;1 isoform identified in *indica* accessions exhibits higher inward Na^+^ currents than those of the *japonica*-predominant isoform. Such a difference was brought about due to a less negative voltage threshold of inward current activation of the *indica*-predominant isoform, which could account for higher root Na^+^ contents and superior salt stress tolerance of the *indica* accessions. In barley, allelic variation in the *HvHKT1* gene is associated with higher salt tolerance of the Tibetan wild barley accessions in comparison with barley cultivars [24]. The *HvHKT1* gene was later found to correspond to *HKT1;1* by a phylogenetic analysis and demonstrated to encode a Na^+^ selective transporter [25]. Knocking-down of *HvHKT1;1*, mediated by barley stripe mosaic virus (BSMV)-induced gene silencing, increased sensitivity to salt stress and accumulation of Na^+^ in both roots and leaves in the barley lines compared with a vector-introduced control line, which demonstrated essentiality of this gene in the mechanism of salt tolerance of barley [25].

According to the gene database of the National Center for Biotechnology Information (NCBI), the *OsHKT1;1* gene produces several mRNA variants in *japonica* rice group. Although evidence of an importance of the *HKT1;1* gene for the salt tolerance mechanism has accumulated as mentioned above, functions and impacts on salt tolerance of these variants are not yet initiated. In this study, we have attempted to identify *OsHKT1;1* variants in a salt tolerant landrace of *indica* rice, Pokkali, and characterized their expression patterns and transport properties by means of quantitative PCR (qPCR) analyses and two electrode voltage clamp experiments using *X. laevis* oocytes. New aspects of transport activity and expression profiles of *OsHKT1;1* variants will be discussed.

## 2. Results

### 2.1. Isolation and Characterization of OsHKT1;1 cDNAs

**Figure 1:**
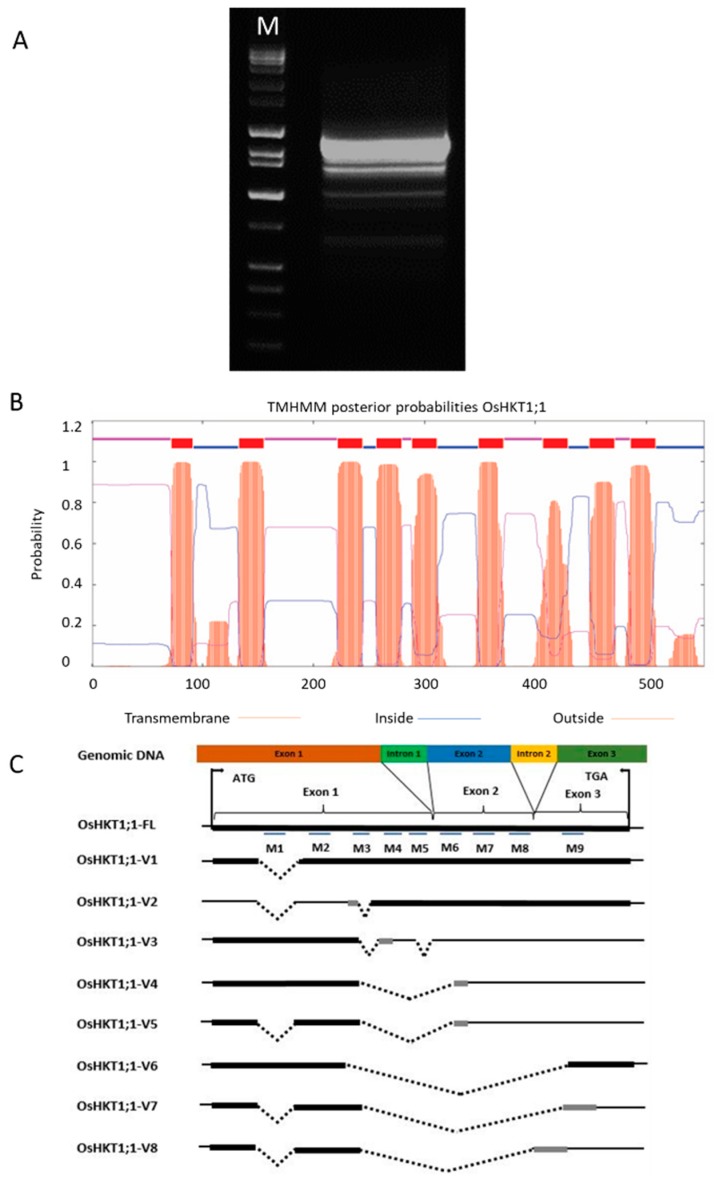
*OsHKT1;1* transcripts identified in Pokkali plants. (**A**) A gel image of RT-PCR produces amplified with primers for *OsHKT1;1*; (**B**) the transmembrane domain of the full-length OsHKT1;1 protein (OsHKT1;1-FL) predicted by TMHMM Server v.2.0. Nine transmembrane domains (M1-M9) were predicted; (**C**) schematic diagrams of OsHKT1;1-FL and its variants. Boxes indicate amino acid regions that are the same as FL (black) or different from FL because of the frame shift (grey). The line “─” indicates non-translated regions, and dot line “┉” indicates missing nucleotide regions (gap) compared with the FL sequence.

Upon the cDNA isolation using primers for the full-length clone, several different fragments were amplified from the first strand population prepared from the whole seedling of a salt tolerant *indica* variety Pokkali (Figure 1A). Sequence analysis indicated the presence of eight splicing variants in addition to the full-length sequence of the *OsHKT1;1* cDNA. The full length *OsHKT1;1* clone (*OsHKT1;1-FL*) is constituted of 1831 nucleotides, which encodes 552 putative amino acid residues. The deduced amino acid sequence was 100% identical to *OsHKT1;1* of a variety Zhenshan 2 [23]. Prediction of the structure of *OsHKT1;1-FL* by the online software TMHMM Server v. 2.0 resulted in nine transmembrane domains as shown in Populus PeHKT1;1 [26] (Figure 1B). Eight *OsHKT1;1* variants, *OsHKT1;1-V1*, *-V2*, *-V3*, *-V4*, *-V5*, *-V6*, *-V7*, and *-V8* cDNAs, were respectively composed of 1716, 1571, 1452, 1181, 1067, 958, 920, and 931 nucleotides, which encode 515, 318, 209, 217, 180, 223, 186, and 176 putative amino acid residues (Figure 1C, Figures S3 and S4).

### 2.2. Expression Analyses of OsHKT1;1 in Shoots and Roots

**Figure 2:**
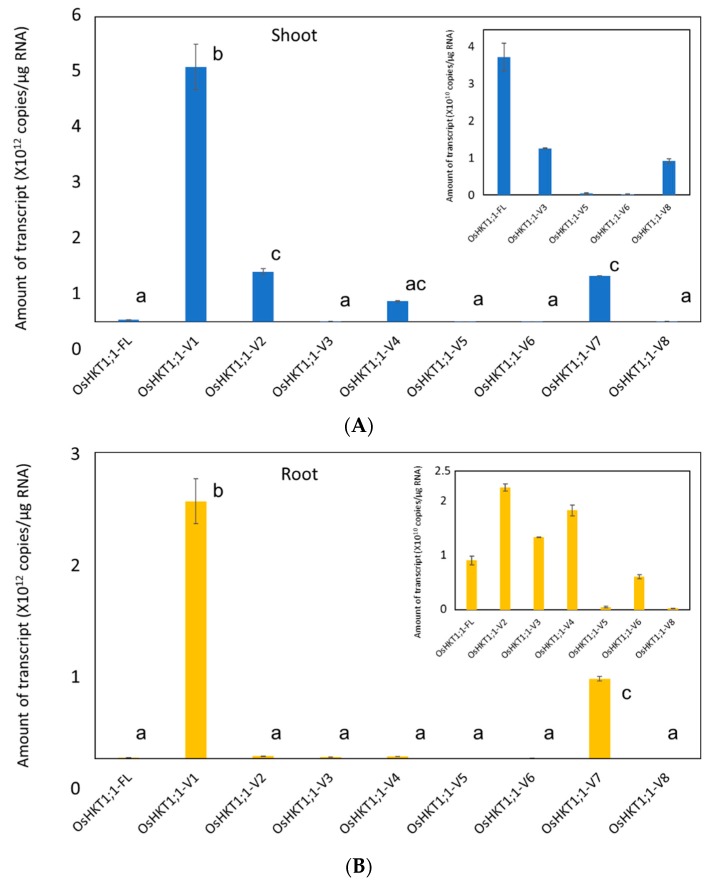
qPCR analyses on *OsHKT1;1* transcripts in Pokkali plants grown under normal growth condition. Expression levels of *OsHKT1;1-FL* and its variants in shoots (**A**) and roots (**B**) of 14 day-old plants were investigated by absolute quantification. Insets; Expanded views of variants showing low expression. Data are means ± SE, *n* = 3 Three independent experiments were performed, and similar results were obtained. Different letters indicate significant differences (*p* < 0.05).

14 days old Pokkali plants were analyzed under a normal growth condition. qPCR analyses revealed that all *OsHKT1;1* transcripts including the *FL* clone were detected in both roots and shoots, and *OsHKT1;1-V1* was the most abundant variant among them (Figure 2). Results also showed that transcript levels of *OsHKT1;1*-V1, -V2, and -V7 were significantly higher than the rests with a tendency of higher accumulation of -*V4* transcripts in shoots. In contrast, *OsHKT1;1*-V1 and -V7 transcripts were more abundant than the others in roots (Figure 2).

**Figure 3:**
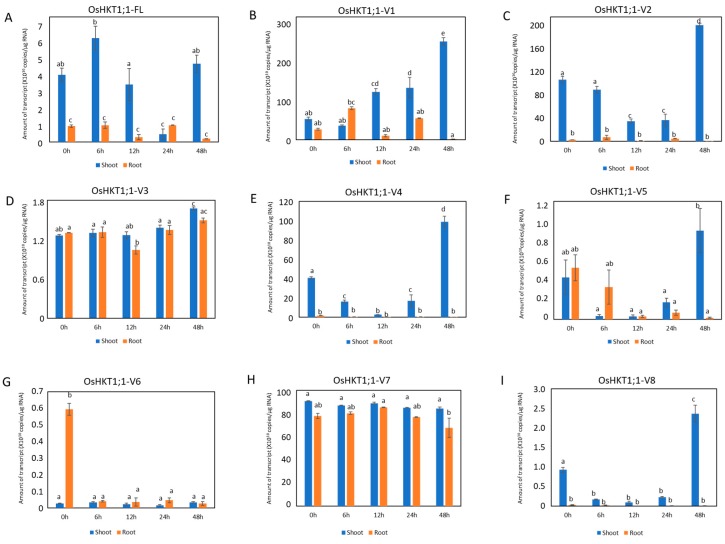
qPCR analyses on *OsHKT1;1* transcripts in Pokkali seedlings grown under salt stressed conditions. Expression levels of *OsHKT1;1-FL* and each variant in shoots and roots were investigated by absolute quantification. 14 day-old Pokkali plants were treated with 100 mM NaCl solution for 0, 6, 12, 24, or 48 h prior to the total RNA extraction. (**A**) *OsHKT1;1-FL*, (**B**) *OsHKT1;1-V1*, (**C**) *OsHKT1;1-V2*, (**D**) *OsHKT1;1-V3*, (**E**) *OsHKT1;1-V4*, (**F**) *OsHKT1;1-V5*, (**G**) *OsHKT1;1-V6*, (**H**) *OsHKT1;1-V7*, (**I**) *OsHKT1;1-V8*. Absolute amounts of transcript (copies/μg RNA) were displayed. Data are means ± SE, *n* = 3. Three independent experiments were performed, and similar results were obtained. Different letters indicate significant differences (*p* < 0.05).

We next investigated the expression of *OsHKT1;1* under salt stress. Time dependent effects of 100 mM NaCl treatments were analyzed by qPCR using samples from shoots and roots of Pokkali plants (Figure 3). Expression of the *OsHKT1;1-FL* transcript in shoots was transiently reduced at 24 h and again upregulated at 48 h of salt stress (Figure 3A). In contrast, its expression in roots was constantly lower (Figure 3A). Among the variants that highly accumulate in shoots, only *OsHKT1;1-V1* showed an increase trend in accordance with the time after salt stress (Figure 3B). On the other hands, *OsHKT1;1-V2* and *-V4* showed a similar trend such that the transcript levels decreased up to 24 h and then increased at 48 h after the stress treatment (Figure 3C,E). *OsHKT1;1-V7* transcripts did not show any characteristic difference in the pattern of its accumulation (Figure 3H). In roots, some fluctuation was found but no notable pattern was observed in most of the transcripts except for *OsHKT1;1-V6*, which showed a significant reduction in the accumulation in response to salt stress (Figure 3G).

### 2.3. Ion Transport Activity of OsHKT1;1-FL and Its Variants

**Figure 4:**
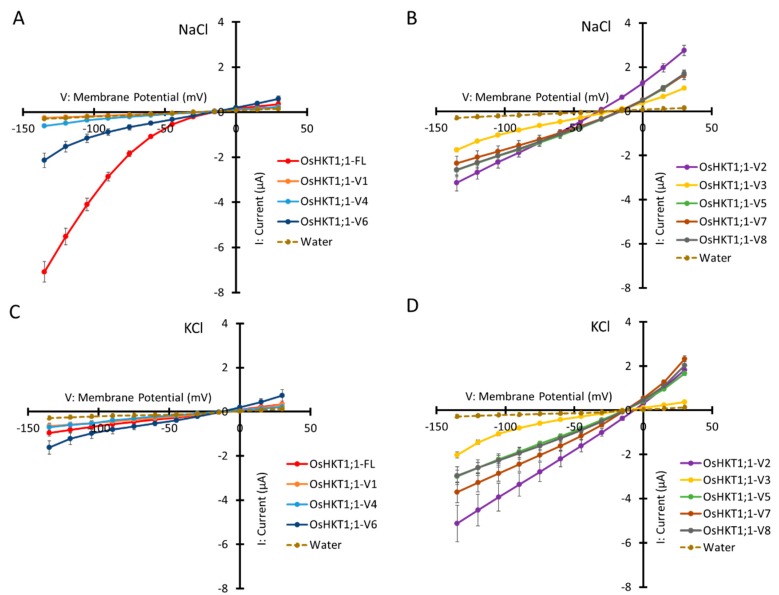
Ion transport activity of OsHKT1;1-FL and its variants. Two electrode voltage clamp experiments using *Xenopus laevis* oocytes were conducted. (**A,C**) Current-voltage relationships from oocytes expressing OsHKT1;1-FL, -V1, -V4, -V6, and water-injection control. (**B,D**) Current-voltage relationships from oocytes expressing OsHKT1;1-V2, -V3, -V5, -V7, -V8, and water-injection control. External solution contains 96 mM NaCl (**A,B**) or 96 mM KCl (**B,D**). All external solutions contain, as background elements, 1.8 mM CaCl_2_, 1.8 mM MgCl_2_, 1.8 mM mannitol, and 10 mM HEPES (pH 7.5 with Tris). Water was injected as a negative control. Data represent means ± SE, *n* = 18–24, from three independent experiments performed on different oocytes batches.

*X. laevis* oocytes were injected with cRNAs of *OsHKT1;1-FL* and its variants, and two electrode voltage clamp (TEVC) experiments were performed. The results exhibited marked differences in ion transport properties of OsHKT1;1 transporters in Na^+^- or K^+^-containing solutions (Figure 4). As has been reported previously [15], OsHKT1;1-FL mediated inward rectifying currents in the presence of 96 mM Na^+^ (Figure 4A). We found that OsHKT1;1-V6 also showed a similar inward rectifying feature in the same solution although elicited currents were smaller than OsHKT1;1-FL (Figure 4A). OsHKT1;1-V1 and -V4 showed small currents similar to water-injected control oocytes (Figure 4A). In contrast, OsHKT1;1-V2, -V3, -V5, -V7, -V8 mediated Na^+^ currents in a bidirectional transport manner depending on the membrane voltage (Figure 4B).

Ion transport activity of OsHKT1;1 transporters was also investigated in the presence of 96 mM K^+^. Only small currents comparable to water-injected oocytes were observed in oocytes expressing OsHKT1;1-FL, -V1 and -V4 (Figure 4C). In OsHKT1;1-V6-expressing oocytes, weak currents, which were slightly higher than water-injected control, were observed with weak rectification. (Figure 4C). Interestingly, expression of OsHKT1;1-V2, -V3, -V5, -V7, and -V8 elicited more robust currents in the K^+^ bath solution (Figure 4D).

## 3. Discussion

Under salt stress several Na^+^ transporters play an essential role in Na^+^ tolerance in plants. Among them, HKT1s have been shown to have a crucial role in both mono and dicotyledonous plants in the tolerance to salinity stress [3,27].

Four distinct sequences of *OsHKT1;1* transcripts in *japonica* rice have been reported (cation transporter HKT4-like: https://www.ncbi.nlm.nih.gov/gene/?term=LOC9266695). In the present study, several splice variants of *OsHKT1;1* were detected in a salt-tolerant *indica* rice Pokkali (Figure 1). Among the previously registered *OsHKT1;1* transcripts from *japonica* rice, the XM_015778164.2 (isoform X1) is the most identical to *OsHKT1;1-FL* isolated from Pokkali in the present study and corresponds to the *japonica OsHKT1;1* clone, the product of which has been well characterized by TEVC experiments [15,23]. In fact, OsHKT1;1-FL from Pokkali was 100% identical to the OsHKT1;1 isoform found in a variety Zhenshan 2, which has been suggested to contribute to conferring more salt tolerance to *indica* rice accessions [23]. The XM_015778165.2 (isoform X2) and the XM_01577817.1 (isoformX4) were respectively the most identical to *OsHKT1;1-V1* and *-V2* clones in the present study (Figure 1C). The XM_015778166.2 (isoform X3), however, did not match any variants isolated in the present study. Therefore, cDNA clones of *OsHKT1;1-V3*, *-V4*, *-V5*, *-V6*, *-V7*, and *-V8* were found only in Pokkali thus far.

Na^+^ exclusion mechanisms mediated by HKT1 transporters has been well studied in several plant species. *Arabidopsis* has only one *HKT* gene, *AtHKT1;1*, which was demonstrated to show passive Na^+^ transport properties under salt stress [11,12]. *AtHKT1;1* is mainly expressed in xylem parenchyma cells and *AtHKT1;1*-mediated unloading of Na^+^ at the xylem was proposed to be a major physiological role of *AtHKT1;1* in the mechanism of salt tolerance in *Arabidopsis* [5,18]. In addition, expression of *AtHKT1;1* in the phloem of leaves was also reported [28] and *AtHKT1;1*-involved Na^+^ recirculation from shoots to roots via the phloem, which contributes to reducing Na^+^ accumulation in leaves and thus tolerance to salt stress, was also proposed [9,18,28]. Salt tolerance QTL analyses using durum wheat have revealed that Na^+^ exclusion from leaves is an essential process to cope with salt stress and governed by two loci *Nax1* and *Nax2* (*Na^+^ exclusion 1 and 2*) [29,30]. Subsequent studies suggested that causal genes for *Nax1* and *Nax2* loci are respectively *TmHKT1;4-A2* and *TmHKT1;5*, both of which encode a Na^+^ selective transporter [1,9,31,32]. Evidence for important contribution of *HKT1;4* and *HKT1;5* genes in rice to salt stress tolerance also accumulated. *OsHKT1;5* encoding a Na^+^ selective transporter, which was suggested to be a causal gene for the *SKC1* (*Shoot K^+^ Content 1*) locus identified in a salt tolerance QTL analysis of rice, has been indicated to protect leaves including the newest expanding one presumably through Na^+^ unloading from the xylem of roots and sheaths and the phloem at the basal node under salt stress [14,15]. A Na^+^ selective transporter *OsHKT1;4* in rice was proposed to function in Na^+^ exclusion from leaves and stems under salt stress at the reproductive growth stage [17]. In addition to these *OsHKT1* genes, *OsHKT1;1* has been reported to play a crucial role in Na^+^ exclusion from shoots in rice [21]. Predominant expression of *OsHKT1;1* in the phloem evokes possible involvement of OsHKT1;1 in the Na^+^ recirculation mechanism from shoots to roots [21], however, detailed physiological functions of OsHKT1;1 is yet to be elucidated. Furthermore, more recently, a genetic study using diverse rice accessions provided evidence that *indica* accessions exhibit higher Na^+^ contents in roots than those in *japonica* accessions upon a moderate salt stress, which is regulated by the locus *Root Na^+^ Content 4 (RNC4)* [23]. The study further indicated that a strong candidate for the causal gene of the *RNC4* appears to be *OsHKT1;1* and that an *OsHKT1;1* isoform found in *indica* accessions exhibits a higher Na^+^ transport activity with a less negative voltage threshold of inward-rectifying current activation in comparison with OsHKT1;1 in *japonica* accessions. Based on the observation, the study suggested that such a difference in the function of the OsHKT1;1 isoform contribute to higher salt tolerance of *indica* accessions.

As a matter of fact, OsHKT1;1-FL identified in this study was indeed 100% identical to the OsHKT1;1 isoform identified by Campbell et al. [19,23]. However, functions and roles of *OsHKT1;1* variants have not been investigated so far. As shown in Figure 4A,B, OsHKT1;1-V2, -V3, -V5, -V6, -V7, and -V8, which were expressed in *X. laevis* oocytes, interestingly, elicited more robust currents in comparison with water-injected control oocytes in the presence of 96 mM Na^+^. Surprisingly, those variants also mediated robust currents in a bath solution containing 96 mM K^+^ (Figure 4C, D). The selectivity of HKT transporters for Na^+^ and K^+^ has been reported to be largely dependent on a key amino acid residue, serine (S) or glycine (G), at the key position in the putative pore-forming region (p-loop) ([9] and references therein). The key amino acid position is relevant to the GYG motif of a shaker K^+^ channel, which is an essential factor for the K^+^ selectivity ([9] and references therein). HKT transporters in general retain four p-loop regions and either S or G in the first p-loop can be consistent with its transport property, that is, a Na^+^ selective type (subfamily I) or a Na^+^-K^+^ co-transport type (subfamily II), respectively [5,9]. OsHKT1;1 retains the S residue at the key position of the first p-loop, which lies in the position of 104–121 amino acids of OsHKT1;1-FL. Except for OsHKT1;1-V2 that retains the rest of three p-loop regions, all other variants kept the first p-loop. Elucidating how those shorter variants can form a mature ion transporter and these subfamily 1-derived variants possibly mediates K^+^ transport will be an important subject to address in the future.

Wang et al. [21] reported approximately 3- to 5-fold increases in the *OsHKT1;1* expression in 12 h after a salt stress in shoots, while no difference in its expression was observed in roots. In the present study, the level of *OsHKT1;1-FL* mRNA did not show a significant difference, but only *OsHKT1;1-V1* mRNA significantly increased in shoots 12 h after the imposition of salt stress (Figure 3). Note however that, looking at 48 h after salt stress, levels of *OsHKT1;1-V2*, *-V4*, *-V5*, and *-V8* mRNA were significantly increased (Figure 3). Our qPCR analyses also revealed that the *OsHKT1;1-V1* mRNA was the most abundant in both roots and shoots among the variants identified (Figure 2). However, V1 showed no transport activity for Na^+^ and K^+^ in *X. laevis* oocytes (Figure 4). Increases in the *OsHKT1;1*-*V1* mRNA after salt stress in shoots (Figure 3B) might have competitive effects on the function of other variants/FL. In addition, OsHKT1;1-V2 and -V7 might also be an interesting target for further research as both variants mediated bidirectional currents stimulated by Na^+^-and K^+^ in oocytes and their expression levels in Pokkali tissues were relatively high compared with *OsHKT1;1-FL* (Figure 2, Figure 3, and Figure 4). These results imply that not only OsHKT1;1-FL but also other variants could carry out some role in Pokkali plants under salt stress.

Pokkali OsHKT1;1-FL in the present study showed an inward-rectifying characteristic in Na^+^ transport as previously reported [15,23]. In contrast, OsHKT1;1-V1 showed no ion transport activity probably because of the loss of the M1 domain from the full-length sequence. However, the function of M1 seems not to be simple as OsHKT1:1-V2 that lacks both M1 and M2 domains showed ion transport activity (Figure 4), suggesting a role of M2 in cancelling the negative influence of the lack of M1 on the activity. Shorter variants suggest complex interactions between M1 and M2 domains and/or latter domains. It should be noted however that possibilities of different rates of translation and/or protein turnover among *OsHKT1;1* variants were not taken into account in electrophysiological characterization by TEVC experiments in this study. Therefore, such aspects need to be considered in future TEVC experiments that will be performed to characterize *OsHKT1;1* variants in more details. Molecular mechanisms of domain interactions and regulation in the ion transport activity and ion selectivity should also be revealed in future studies.

## 4. Materials and Methods

### 4.1. Plant Material and Growth Condition

A salt tolerant landrace of rice, Pokkali, (*Oryza sativa* L. ssp. *indica*) was used in this study. Seeds were sterilized two times by using 50% bleach for 10 min and then washed with sterilized water for 5 times. For germination, ten seeds were placed on a mesh in a sterilized pot with 20 mL of 1 mM CaSO_4_. Plants were grown in a controlled environment growth chamber. The temperature was maintained at 28 °C and 25 °C in 12 h day (250 μmol m^−2^ s^−1^ illumination) and 12 h night, respectively. Five-day-old seedlings were transferred to 3.5-liter pots and grown hydroponically with the solutions containing 4 mM KNO_3_, 1 mM NH_4_H_2_PO_4_.2H_2_O, 1 mM CaCl_2_.2H_2_O, 1 mM MgSO_4_.7H_2_O, and micronutrients (1 ppm Fe, 0,5 ppm B, 0.5 ppm Mn, 0.05 ppm Zn, 0.02 ppm Cu, and 0.01 ppm Mo). The pH of the nutrient solution was adjusted to 5.5 with NaOH. 9-day old plants were used for cDNA isolation. For the gene expression study, 14 day-old plants were subjected to 100 mM NaCl stress for 0 h (control), 6 h, 12 h, 24 h, and 48 h. Roots and shoots were then collected separately at different time points.

### 4.2. Extraction of DNA and RNA, and cDNA Synthesis

Pokkali genomic DNA was extracted from young leaves using a Cica Geneus DNA Prep Kit for Plants (Kanto, Japan) following the manufacturer’s instructions. Total RNA was extracted from root or shoot samples using a RNeasy Plant Mini Kit (Qiagen, Hilden, Germany) and then treated with RNase-free DNase I (Ambion, Austin, TX, USA). The quality and integrity of RNA samples were determined by a NanoDrop ND-1000 Spectrophotometer (Nanodrop, Wilmington, DE, USA). The first strand coding DNA (cDNA) was synthesized from total RNA using ReverTra Ace qPCR RT Master Mix (TOYOBO, Osaka City, Japan), according to the manufacturer’s instructions. *OsHKT1;1* cDNAs was amplified by using *OsHKT1;1* cloning primers (Supplemental Table S1) and confirmed by analyzing the sequence after cloning into the pCR4 topo vector (Invitrogen, Carlsbad, CA, USA).

### 4.3. Secondly Structure Analysis

Transmembrane domains were predicted by the online software TMHMM Server v. 2.0 (http://www.cbs.dtu.dk/services/TMHMM/) applied to Populus PeHKT1;1 [26].

### 4.4. Expression Analysis

Total RNA samples were isolated from shoot and root tissues of 14 day-old plants (control and 100 mM NaCl) and the first-strand cDNA was reverse transcribed with a high capacity cDNA reverse transcription kit (Applied Biosystem, Foster City, CA, USA). The *OsHKT1;1* variants were amplified using specific primers (Supplement Table S1). Amplification of the actin mRNA was used as an internal control as described previously [33]. Absolute quantification was performed in the quantitative real-time PCR analysis using the 7300 real-time PCR machine (Applied Biosystem, Foster City, CA, USA) as described in [34] to analyze the expression level of each *OsHKT1;1* variant. Specific cDNAs were used as a standard to quantify each variant, and the primer information was indicated in the Supplementary Figure S1.

### 4.5. Expression in Xenopus laevis (X. leavis) Oocytes

*OsHKT1;1* cDNAs from Pokkali were subcloned into the pXβG vector between the 5´ and 3´ untranslated regions of the Xenopus β-globin gene. Capped cRNAs were synthesized in vitro from the linearized vector using the mMESSAGE mMACHINE T3 kit (Ambion, Austin, TX, USA). Oocytes were isolated and injected with 50 ng/50 nL of *OsHKT1;1* cRNA solutions or with 50 nL of nuclease-free water (for control oocytes), which were then incubated at 18 °C in modified Barth’s solution (MBS; 88 mM NaCl, 1 mM KCl, 2.4 mM NaHCO_3_, 15 mM Tris-HCl (pH 7.6), 0.3 mM Ca(NO_3_)_2_.4H_2_O, 0.41 mM CaCl_2_.4H_2_O, 0.82 mM MgSO_4_.7H_2_O, 10 μg ml^−1^ sodium penicillin, and 10 μg ml^−1^ streptomycin sulfate) until electrophysiological recordings as described previously [35].

### 4.6. Electrophysiology

Whole oocyte currents were recorded using the two-electrode voltage-clamp (TEVC) technique 1 to 2 d after the cRNA injection. TEVC recordings and data analysis were performed using an Axoclamp 900A amplifier, an Axon Instruments Digidata 1440A, Clampex 10.3, and Clampfit 10.3 (Molecular Devices, San Jose, CA, USA). All bath solutions contained a background of 1.8 mM MgCl_2_, 1.8 mM CaCl_2_, and 10 mM HEPES pH 7.5 with Tris. Monovalent cation was used as chloride salts (chloride concentration constant in each set of solutions). D-mannitol was added when necessary to adjust the osmolality (same osmolality in each set of solutions in the range 200–220 mOsm). Voltage steps (2 sec) were applied from +30 to −135 mV in 15 mV decrements. All experiments were performed at room temperature (20–22 °C).

### 4.7. Statistics

Statistical analyses were performed using IBM SPSS Statistics Desktop for Japan. Significant differences were identified by one-way analysis of variance followed by Tukey HSD (*p* < 0.05).

## Supplementary Materials

The following are available online at https://www.mdpi.com/2223-7747/9/1/16/s1, Table S1: Gene-specific primer pairs used for the cloning and in the real-time PCR experiments, Figure S1: Specific primer position of OsHKT1;1-FL and its eight variant for qPCR assay, Figure S2: Nucleotide sequence alignment of Pokkali OsHKT1;1 genomic DNA and full-length cDNA, Figure S3: Nucleotide sequence alignment of OsHKT1;1-FL and its eight variant, Figure S4; Protein sequence alignment of OsHKT1;1-FL and its eight variant, Figure S5: Expression analyzed with RT-PCR.
