# Supplementary material for: Expression and Ion Transport Activity of Rice OsHKT1;1 Variants
Source: Plants (Basel). 2019 Dec 21;9(1):16. doi: 10.3390/plants9010016 (PMC7020204; doi:10.3390/plants9010016)
Supplement: Supplementary file 1 [file plants-09-00016-s001.pdf]

Electrophysiological Analysis of Rice OsHKT1;1 Variants

Shahin Imran, Tomoaki Horie, Maki Katsuhara

Supplementary Materials: Table S1: Gene-specific primer pairs used for the cloning and in the real-time PCR experiments, Figure S1: Specific primer position of OsHKT1;1-FL and its eight variant for qPCR assay, Figure S2: Nucleotide sequence alignment of Pokkali OsHKT1;1 genomic DNA and full-length cDNA, Figure S3: Nucleotide sequence alignment of OsHKT1;1-FL and its eight variant, Figure S4; Protein sequence alignment of OsHKT1;1-FL and its eight variant, Figure S5: Expression analyzed with RT-PCR.

Supplementary Table S1.
Gene-specific primer pairs used for the cloning and in the real-time PCR experiments.

	5’ → 3’	For full-length cloning	For RT-PCR and real-time RT-PCR
OsHKT1;1-FL	Forward	GTTGAAGAATGCATCCACCAAG	
	Reverse	CTCATTTCAGGATGAACTCCTTG	
OsHKT1;1-FL	Forward		CCCTTTGGTAGTTCAGCTCGTTT
	Reverse		GATGACAGAACTGGAAATGACAG
OsHKT1;1-V1	Forward		AGATGTGTGAATCTCAAGCCCCAG
	Reverse		ATAACCTGTCACTATGCGAACCAA
OsHKT1;1-V2	Forward		CGCAAAACCAGATGCAAGAAATG
	Reverse		CCTCACATGCAAATGTTTGTAGCC
OsHKT1;1-V3	Forward		GCTACAAACATTTGCATGTACCTTCC
	Reverse		AACTTCTGGCAACTGTATCCTAGTG
OsHKT1;1-V4	Forward		AAACCAGGTACCTTCCTTCCGAT
	Reverse		GGCAACTGTATCCTAGTGAGTAGC
OsHKT1;1-V5	Forward		CCAGATGTGTGAATCTCAAGCCCC
	Reverse		CATCGGAAGGAAGGTACCTGGTTTT
OsHKT1;1-V6	Forward		ATGCTTCCTACGGGTTCGTCGGCAGGT
	Reverse		TTATTAGGAGCAGATACGAATGGC
OsHKT1;1-V7	Forward		CCCTCACTAAAGGGAACAAAAGC
	Reverse		GGGGCTTGAGATTCACACATCTG
OsHKT1;1-V8	Forward		GTCGCAAAACCAGAAAGGAAGTCAA
	Reverse		GGATAACTATCAGCTTACCCTCCTC
Actin	Forward		CTAGCCTCACTCAGCACTTT
	Reverse		CGTGGAGATCAGAAGCAC

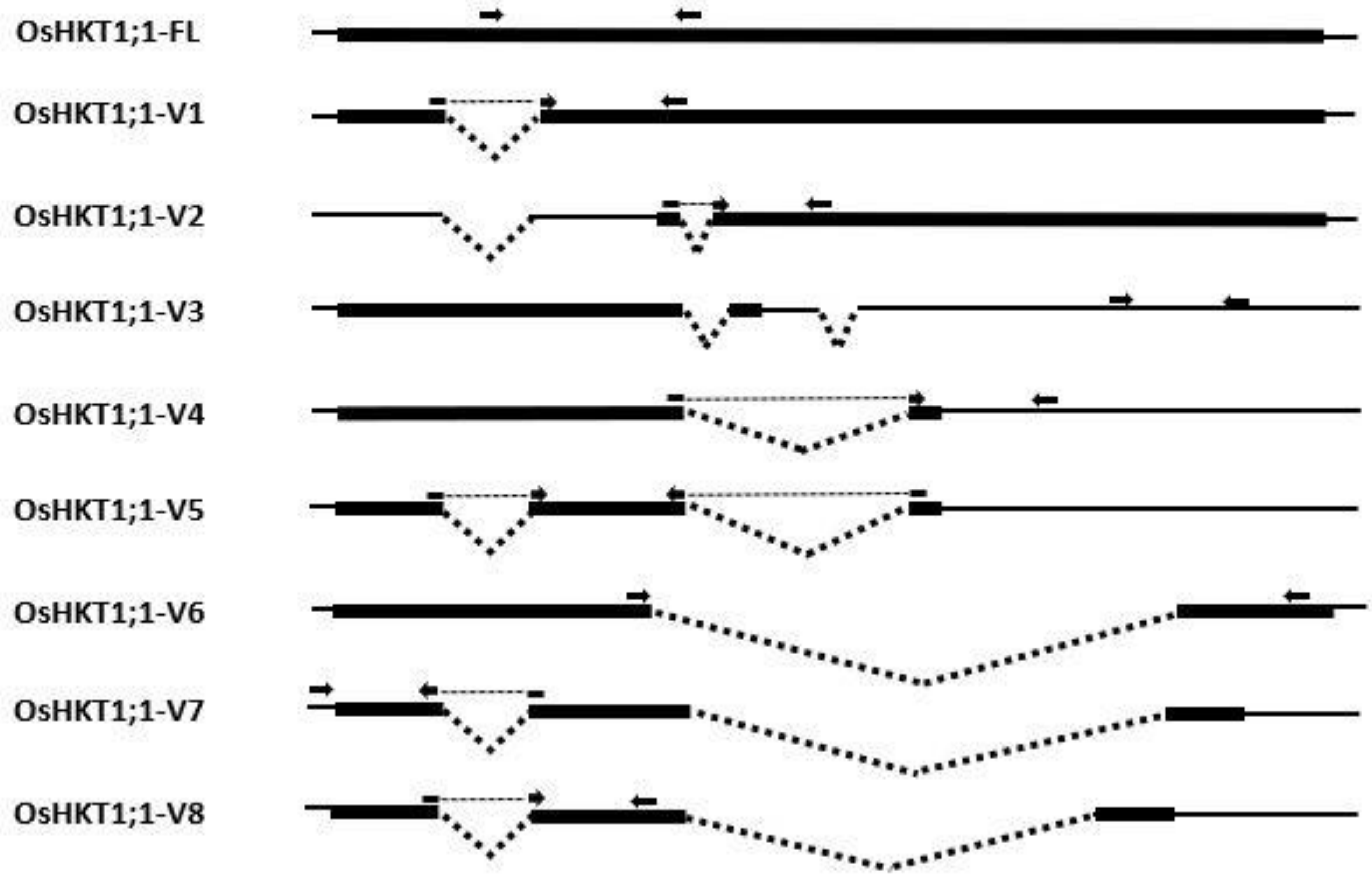

Supplementary Figure S1.
Specific primer position of OsHKT1;1-FL and its eight variant for qPCR assay. Right arrows indicate forward primers, and left arrows indicate reverse primer.

```
Pokkali OsHKT1;1 Genomic DNA    0 ------------------------------------------------------------ 0
OsHKT1;1-FL                     1 GGGCCCCCTCGAGGTCGACGGTATCGATAAGCTTGCTTGTTCTTTTTGCAGAAGCTCAGA 60

Pokkali OsHKT1;1 Genomic DNA    1 ----------------------------------------------------AACTTGAA 8
OsHKT1;1-FL                    61 ATAAACGCTCAACTTTGGCAGAACCATGCATCCACCAAGTTTAGTGCTAGATACCTTGAA 120

Pokkali OsHKT1;1 Genomic DNA    9 GCGTATCAA-CTATACATAGCCATGAAGCTCCTGTTACCGAATTCGGAGGTGCTTCGGAT 67
OsHKT1;1-FL                   121 GCGTATCAAACTATACATAGCCATGAAGCTCCTGTTACCGAATTCGGAGGTGCTTCGGAT 180

Pokkali OsHKT1;1 Genomic DNA   68 CTATTGGGAGAAAGCTCAGCATCTCTGTGGGTTCCTGTCCATGAAGCTCATTTCCAGAGC 127
OsHKT1;1-FL                   181 CTATTGGGAGAAAGCTCAGCATCTCTGTGGGTTCCTGTCCATGAAGCTCATTTCCAGAGC 240

Pokkali OsHKT1;1 Genomic DNA  128 CAGATGTGTGGCAAGTTCTGTCAAACAATCTTACAGTTTTCTGGTTTGCAAAAGTAACCC 187
OsHKT1;1-FL                   241 CAGATGTGTGGCAAGTTCTGTCAAACAATCTTACAGTTTTCTGGTTTGCAAAAGTAACCC 300

Pokkali OsHKT1;1 Genomic DNA  188 TTTGGTAGTTCAGCTCGTTTACTTTGTGATAATCTCATTTGCTGGTTTCCTTGCTCTGAA 247
OsHKT1;1-FL                   301 TTTGGTAGTTCAGCTCGTTTACTTTGTGATAATCTCATTTGCTGGTTTCCTTGCTCTGAA 360

Pokkali OsHKT1;1 Genomic DNA  248 GAATCTCAAGCCCCAGGGTAAGCCAGGTCCAAAGGATTTGGACCTGTTGTTCACCTCTGT 307
OsHKT1;1-FL                   361 GAATCTCAAGCCCCAGGGTAAGCCAGGTCCAAAGGATTTGGACCTGTTGTTCACCTCTGT 420

Pokkali OsHKT1;1 Genomic DNA  308 GTCTACACTTACTGTCTCGAGCATGGCAACAGTAGAGATGGAAGACTTATCTGACAGGCA 367
OsHKT1;1-FL                   421 GTCTACACTTACTGTCTCGAGCATGGCAACAGTAGAGATGGAAGACTTATCTGACAGGCA 480

Pokkali OsHKT1;1 Genomic DNA  368 ACTCTGGGTTCTGATCCTTCTGATGCTAATGGGAGGAGAGGTGTTCACATCAATGCTAGG 427
OsHKT1;1-FL                   481 ACTCTGGGTTCTGATCCTTCTGATGCTAATGGGAGGAGAGGTGTTCACATCAATGCTAGG 540

Pokkali OsHKT1;1 Genomic DNA  428 GCTCTACTTCAACAATGCCAATGCCAACAGAAATGAGAACAGCCAGAGAAGTTTACCTTC 487
OsHKT1;1-FL                   541 GCTCTACTTCAACAATGCCAATGCCAACAGAAATGAGAACAGCCAGAGAAGTTTACCTTC 600

Pokkali OsHKT1;1 Genomic DNA  488 AATCAGCTTGGACATTGAATCCAACAGTCCTGCAAACAATGGGGATCACAAAATTACGGA 547
OsHKT1;1-FL                   601 AATCAGCTTGGACATTGAATCCAACAGTCCTGCAAACAATGGGGATCACAAAATTACGGA 660

Pokkali OsHKT1;1 Genomic DNA  548 ATGTGGCCAATCAGAAGAAACTATGTCGCAAAACCAGGTACAGCAAAACAAAAGCATAAC 607
OsHKT1;1-FL                   661 ATGTGGCCAATCAGAAGAAACTATGTCGCAAAACCAGGTACAGCAAAACAAAAGCATAAC 720

Pokkali OsHKT1;1 Genomic DNA  608 ATATAATCCTTGCGCTGTGTTGGTTCGCATAGTGACAGGTTATTTCGTAGCTACTGTCAT 667
OsHKT1;1-FL                   721 ATATAATCCTTGCGCTGTGTTGGTTCGCATAGTGACAGGTTATTTCGTAGCTACTGTCAT 780

Pokkali OsHKT1;1 Genomic DNA  668 TTCCAGTTCTGTCATCATTATTATTTACTTTTGGATTGATTCAGATGCAAGAAATGTACT 727
OsHKT1;1-FL                   781 TTCCAGTTCTGTCATCATTATTATTTACTTTTGGATTGATTCAGATGCAAGAAATGTACT 840

Pokkali OsHKT1;1 Genomic DNA  728 GAAAAGTAAGGAGATCAGTATGTATACCTTTTGCATCTTCACAGCAGTGTCCTCGTTCGC 787
OsHKT1;1-FL                   841 GAAAAGTAAGGAGATCAGTATGTATACCTTTTGCATCTTCACAGCAGTGTCCTCGTTCGC 900

Pokkali OsHKT1;1 Genomic DNA  788 AAACTGTGGCTTCACGCCACTAAATAGTAACATGCAACCCTTCAGAAAGAACTGGGTCCT 847
OsHKT1;1-FL                   901 AAACTGTGGCTTCACGCCACTAAATAGTAACATGCAACCCTTCAGAAAGAACTGGGTCCT 960

Pokkali OsHKT1;1 Genomic DNA  848 TTTGCTCCTAGTGATCCCGCAGATTCTAGCAGGCAATACCTTGTTTTCACCACTCTTGCG 907
OsHKT1;1-FL                   961 TTTGCTCCTAGTGATCCCGCAGATTCTAGCAGGCAATACCTTGTTTTCACCACTCTTGCG 1020

Pokkali OsHKT1;1 Genomic DNA  908 GCTATGCGTATGGGTTTTGGGGAAGGTCAGTGGAAAAGCAGAGTATGCTTACATCCTTCA 967
OsHKT1;1-FL                  1021 GCTATGCGTATGGGTTTTGGGGAAGGTCAGTGGAAAAGCAGAGTATGCTTACATCCTTCA 1080

Pokkali OsHKT1;1 Genomic DNA  968 GCATCCTGGGGAGACTGGCTACAAACATTTGCATGTGAGGAGAAATTCTGTTTACATTGT 1027
OsHKT1;1-FL                  1081 GCATCCTGGGGAGACTGGCTACAAACATTTGCATGTGAGGAGAAATTCTGTTTACATTGT 1140

Pokkali OsHKT1;1 Genomic DNA 1028 TCTGAGTGTTACTGGACTGATTCTACTGCAAGTAATGTTTATTTGCTCGTTTGAGTGGAA 1087
OsHKT1;1-FL                  1141 TCTGAGTGTTACTGGACTGATTCTACTGCAAGTAATGTTTATTTGCTCGTTTGAGTGGAA 1200

Pokkali OsHKT1;1 Genomic DNA 1088 CTCAGAGAGCTTAGAGGGAATGAATTGGTTACAGAAATTGGTAGGATTATTATTCCAGAG 1147
OsHKT1;1-FL                  1201 CTCAGAGAGCTTAGAGGGAATGAATTGGTTACAGAAATTGGTAGGATTATTATTCCAGAG 1260

Pokkali OsHKT1;1 Genomic DNA 1148 TGTTAATACAAGACAAGCCGGTGAATCTATCCTTGATATATCAACACTTTCTCCATCAAC 1207
OsHKT1;1-FL                  1261 TGTTAATACAAGACAAGCCGGTGAATCTATCCTTGATATATCAACACTTTCTCCATCAAC 1320

Pokkali OsHKT1;1 Genomic DNA 1208 TCTGTTGCTATTTGCAGTCGTCATGTTAGTATCTCCTCCAAACCCTGTCTTCTAATGAAT 1267
OsHKT1;1-FL                  1321 TCTGTTGCTATTTGCAGTCGTCATGT---------------------------------- 1346

Pokkali OsHKT1;1 Genomic DNA 1268 TTAACTAAATCAGTAGAACACTTAGATTAGTTATTACTGGAACATAACAAATTATCAGGA 1327
OsHKT1;1-FL                  1346 ------------------------------------------------------------ 1346

Pokkali OsHKT1;1 Genomic DNA 1328 GAAACTGTTGGAAAATTTGATAGCTTCATCATTTATAACTTTGAAGATTACTAAACACAT 1387
OsHKT1;1-FL                  1346 ------------------------------------------------------------ 1346

Pokkali OsHKT1;1 Genomic DNA 1388 GCATTCATGACCACTGCAGCTTGCTAGGTTTTATCGATTTTATACGTCACCGAAATAGTA 1447
OsHKT1;1-FL                  1346 ------------------------------------------------------------ 1346

Pokkali OsHKT1;1 Genomic DNA 1448 GTTATGAACTTCAAATCTGCATCTAAATAACTACTGTTGAATAGAGAAAGTGAGACTACT 1507
OsHKT1;1-FL                  1346 ------------------------------------------------------------ 1346

Pokkali OsHKT1;1 Genomic DNA 1508 TTGAACAAATAGGACAGAACTTCTGACAAGGGTTTAAATGAAAAGTAGGCAGCATACAAC 1567
OsHKT1;1-FL                  1346 ------------------------------------------------------------ 1346

Pokkali OsHKT1;1 Genomic DNA 1568 ATGTTTTATGTGGAACAGCATGTTTCCTACTCCTTGTTAACAGATAATCAACTTGCTTGC 1627
OsHKT1;1-FL                  1346 ------------------------------------------------------------ 1346

Pokkali OsHKT1;1 Genomic DNA 1628 AGGTACCTTCCTTCCGATGCTTCATTTCTCACTGCCAATGCTGATAACCAGCCCTTGACA 1687
OsHKT1;1-FL                  1347 ----ACCTTCCTTCCGATGCTTCATTTCTCACTGCCAATGCTGATAACCAGCCCTTGACA 1402

Pokkali OsHKT1;1 Genomic DNA 1688 GATAAAAAGACAAACTCGATTAGCAGAGCACTGTGGAGGAATTTTACCGTTAACAAGCTT 1747
OsHKT1;1-FL                  1403 GATAAAAAGACAAACTCGATTAGCAGAGCACTGTGGAGGAATTTTACCGTTAACAAGCTT 1462

Pokkali OsHKT1;1 Genomic DNA 1748 TCTTGTTTAGCAATGTTCACATTCCTGGCATGCATAACAGAAAGGAAGTCAATTTCTTCT 1807
OsHKT1;1-FL                  1463 TCTTGTTTAGCAATGTTCACATTCCTGGCATGCATAACAGAAAGGAAGTCAATTTCTTCT 1522

Pokkali OsHKT1;1 Genomic DNA 1808 GATCCACTAAATTTCAACATCTTCAGCATAGTTTTTGAGATAATCAGGCAAGTTCACATC 1867
OsHKT1;1-FL                  1523 GATCCACTAAATTTCAACATCTTCAGCATAGTTTTTGAGATAATCAG------------- 1569

Pokkali OsHKT1;1 Genomic DNA 1868 CTTTTTTTTTGTCTGCCCATAACAGAAACTATGGCTACATCAAAATTACATCACTGAAAA 1927
OsHKT1;1-FL                  1569 ------------------------------------------------------------ 1569

Pokkali OsHKT1;1 Genomic DNA 1928 TCCGAATTGCCTAGTATGATCACTAAGAACCACTGAACCAGCATAATTAAATTTGCAAGA 1987
OsHKT1;1-FL                  1569 ------------------------------------------------------------ 1569

Pokkali OsHKT1;1 Genomic DNA 1988 ACATATCAAATCTACGACCTGTCATTTTTTTTTCTTTTTTGGTTGCTTACAAGCAAACAG 2047
OsHKT1;1-FL                  1569 ------------------------------------------------------------ 1569

Pokkali OsHKT1;1 Genomic DNA 2048 AAATAACGCCATTTTATTTCCACAGCGCTTTCGGCAATGTAGGCTACTCACTAGGATACA 2107
OsHKT1;1-FL                  1570 -------------------------CGCTTTCGGCAATGTAGGCTACTCACTAGGATACA 1604

Pokkali OsHKT1;1 Genomic DNA 2108 GTTGCCAGAAGTTGTTGAAGCCTGATGCCACTTGCAAAGATGCTTCCTACGGGTTCGTCG 2167
OsHKT1;1-FL                  1605 GTTGCCAGAAGTTGTTGAAGCCTGATGCCACTTGCAAAGATGCTTCCTACGGGTTCGTCG 1664

Pokkali OsHKT1;1 Genomic DNA 2168 GCAGGTGGACCGAGGAGGGTAAGCTGATAGT----------------------------- 2198
OsHKT1;1-FL                  1665 GCAGGTGGACCGAGGAGGGTAAGCTGATAGTTATCCTGGTGATGTTCCTGGGGAGGCTCA 1724

Pokkali OsHKT1;1 Genomic DNA 2198 ------------------------------------------------------------ 2198
OsHKT1;1-FL                  1725 AGGAGTTCATCCTGAAATGAAGCGGCCGCGACGGTACCACTAAACCAGCCTCAAGAACAC 1784

Pokkali OsHKT1;1 Genomic DNA 2198 -----------------------------------------------              2198
OsHKT1;1-FL                  1785 CCGAATGGAGTCTCTAAGCTACATAATACCAACTTACACTTACAAAA              1831
```

Supplement Figure S2.

Nucleotide sequence alignment of Pokkali OsHKT1;1 genomic DNA and full-length cDNA. GENETYX ver. 13 was used for exon-intron analysis.

```
OsHKT1;1-FL     0 ------------------------------------------------------------ 0
OsHKT1;1-V1     0 ------------------------------------------------------------ 0
OsHKT1;1-V2     0 ------------------------------------------------------------ 0
OsHKT1;1-V3     0 ------------------------------------------------------------ 0
OsHKT1;1-V4     0 ------------------------------------------------------------ 0
OsHKT1;1-V5     0 ------------------------------------------------------------ 0
OsHKT1;1-V6     1 AACAGCTATGACCATGATACGCCAAGCGCGCAATTACCCTCACTAAAGGGAACAAAAGCT 60
OsHKT1;1-V7     1 -----------------------------------ACCCTCACTAAAGGGAACAAAAGCT 25
OsHKT1;1-V8     0 ------------------------------------------------------------ 0

OsHKT1;1-FL     1 ---------GGGCCCCCTCGAGGTCGACGGTATCGATAAGCTTGCTTGTTCTTTTTGCAG 51
OsHKT1;1-V1     1 -------------CCCCTCGAGGTCGACGGTATCGATAAGCTTGCTTGTTCTTTTTGCAG 47
OsHKT1;1-V2     1 -----------CCCCCCTCGAGGTCGACGGTATCGATAAGCTTGCTTGTTCTTTTTGCAG 49
OsHKT1;1-V3     1 ---------GGGCCCCCTCGAGGTCGACGGTATCGATAAGCTTGCTTGTTCTTTTTGCAG 51
OsHKT1;1-V4     1 ------------CCCCCTCGAGGTCGACGGTATCGATAAGCTTGCTTGTTCTTTTTGCAG 48
OsHKT1;1-V5     1 ---------------CCTCGAGGTCGACGGTATCGATAAGCTTGCTTGTTCTTTTTGCAG 45
OsHKT1;1-V6    61 GGGTACCGGGCCCCCCCTCGAGGTCGACGGTATCGATAAGCTTGCTTGTTCTTTTTGCAG 120
OsHKT1;1-V7    26 GGGTACCGGGCCCCCCCTCGAGGTCGACGGTATCGATAAGCTTGCTTGTTCTTTTTGCAG 85
OsHKT1;1-V8     1 --------------CCCTCGAGGTCGACGGTATCGATAAGCTTGCTTGTTCTTTTTGCAG 46
                                                   Start codon
OsHKT1;1-FL    52 AAGCTCAGAATAAACGCTCAACTTTGGCAGAACCATGCATCCACCAAGTTTAGTGCTAGA 111
OsHKT1;1-V1    48 AAGCTCAGAATAAACGCTCAACTTTGGCAGAACCATGCATCCACCAAGTTTAGTGCTAGA 107
OsHKT1;1-V2    50 AAGCTCAGAATAAACGCTCAACTTTGGCAGAACCATGCATCCACCAAGTTTAGTGCTAGA 109
OsHKT1;1-V3    52 AAGCTCAGAATAAACGCTCAACTTTGGCAGAACCATGCATCCACCAAGTTTAGTGCTAGA 111
OsHKT1;1-V4    49 AAGCTCAGAATAAACGCTCAACTTTGGCAGAACCATGCATCCACCAAGTTTAGTGCTAGA 108
OsHKT1;1-V5    46 AAGCTCAGAATAAACGCTCAACTTTGGCAGAACCATGCATCCACCAAGTTTAGTGCTAGA 105
OsHKT1;1-V6   121 AAGCTCAGAATAAACGCTCAACTTTGGCAGAACCATGCATCCACCAAGTTTAGTGCTAGA 180
OsHKT1;1-V7    86 AAGCTCAGAATAAACGCTCAACTTTGGCAGAACCATGCATCCACCAAGTTTAGTGCTAGA 145
OsHKT1;1-V8    47 AAGCTCAGAATAAACGCTCAACTTTGGCAGAACCATGCATCCACCAAGTTTAGTGCTAGA 106

OsHKT1;1-FL   112 TACCTTGAAGCGTATCAAACTATACATAGCCATGAAGCTCCTGTTACCGAATTCGGAGGT 171
OsHKT1;1-V1   108 TACCTTGAAGCGTATCAAACTATACATAGCCATGAAGCTCCTGTTACCGAATTCGGAGGT 167
OsHKT1;1-V2   110 TACCTTGAAGCGTATCAAACTATACATAGCCATGAAGCTCCTGTTACCGAATTCGGAGGT 169
OsHKT1;1-V3   112 TACCTTGAAGCGTATCAAACTATACATAGCCATGAAGCTCCTGTTACCGAATTCGGAGGT 171
OsHKT1;1-V4   109 TACCTTGAAGCGTATCAAACTATACATAGCCATGAAGCTCCTGTTACCGAATTCGGAGGT 168
OsHKT1;1-V5   106 TACCTTGAAGCGTATCAAACTATACATAGCCATGAAGCTCCTGTTACCGAATTCGGAGGT 165
OsHKT1;1-V6   181 TACCTTGAAGCGTATCAAACTATACATAGCCATGAAGCTCCTGTTACCGAATTCGGAGGT 240
OsHKT1;1-V7   146 TACCTTGAAGCGTATCAAACTATACATAGCCATGAAGCTCCTGTTTCCGAATTCGGAGGT 205
OsHKT1;1-V8   107 TACCTTGAAGCGTATCAAACTATACATAGCCATGAAGCTCCTGTTACCGAATTCGGAGGT 166

OsHKT1;1-FL   172 GCTTCGGATCTATTGGGAGAAAGCTCAGCATCTCTGTGGGTTCCTGTCCATGAAGCTCAT 231
OsHKT1;1-V1   168 GCTTCGGATCTATTGGGAGAAAGCTCAGCATCTCTGTGGGTTCCTGTCCATGAAGCTCAT 227
OsHKT1;1-V2   170 GCTTCGGATCTATTGGGAGAAAGCTCAGCATCTCTGTGGGTTCCTGTCCATGAAGCTCAT 229
OsHKT1;1-V3   172 GCTTCGGATCTATTGGGAGAAAGCTCAGCATCTCTGTGGGTTCCTGTCCATGAAGCTCAT 231
OsHKT1;1-V4   169 GCTTCGGATCTATTGGGAGAAAGCTCAGCATCTCTGTGGGTTCCTGTCCATGAAGCTCAT 228
OsHKT1;1-V5   166 GCTTCGGATCTATTGGGAGAAAGCTCAGCATCTCTGTGGGTTCCTGTCCATGAAGCTCAT 225
OsHKT1;1-V6   241 GCTTCGGATCTATTGGGAGAAAGCTCAGCATCTCTGTGGGTTCCTGTCCATGAAGCTCAT 300
OsHKT1;1-V7   206 GCTTCGGATCTATTGGGAGAAAGCTCAGCATCTCTGTGGGTTCCTGTCCATGAAGCTCAT 265
OsHKT1;1-V8   167 GCTTCGGATCTATTGGGAGAAAGCTCAGCATCTCTGTGGGTTCCTGTCCATGAAGCTCAT 226

OsHKT1;1-FL   232 TTCCAGAGCCAGATGTGTGGCAAGTTCTGTCAAACAATCTTACAGTTTTCTGGTTTGCAA 291
OsHKT1;1-V1   228 TTCCAGAGCCAGATGTGT------------------------------------------ 245
OsHKT1;1-V2   230 TTCCAGAGCCAGATGTGTG----------------------------------------- 248
OsHKT1;1-V3   232 TTCCAGAGCCAGATGTGTGGCAAGTTCTGTCAAACAATCTTACAGTTTTCTGGTTTGCAA 291
OsHKT1;1-V4   229 TTCCAGAGCCAGATGTGTGGCAAGTTCTGTCAAACAATCTTACAGTTTTCTGGTTTGCAA 288
OsHKT1;1-V5   226 TTCCAGAGCCAGATGTGT------------------------------------------ 243
OsHKT1;1-V6   301 TTCCAGAGCCAGATGTGTGGCAAGTTCTGTCAAACAATCTTACAGTTTTCTGGTTTGCAA 360
OsHKT1;1-V7   266 TTCCAGAGCCAGATGTGT------------------------------------------ 283
OsHKT1;1-V8   227 TTCCAGAGCCAGATGTGT------------------------------------------ 244
                                                   M1
OsHKT1;1-FL   292 AAGTAACCCTTTGGTAGTTCAGCTCGTTTACTTTGTGATAATCTCATTTGCTGGTTTCCT 351
OsHKT1;1-V1   245 ------------------------------------------------------------ 245
OsHKT1;1-V2   248 ------------------------------------------------------------ 248
OsHKT1;1-V3   292 AAGTAACCCTTTGGTAGTTCAGCTCGTTTACTTTGTGATAATCTCATTTGCTGGTTTCCT 351
OsHKT1;1-V4   289 AAGTAACCCTTTGGTAGTTCAGCTCGTTTACTTTGTGATAATCTCATTTGCTGGTTTCCT 348
OsHKT1;1-V5   243 ------------------------------------------------------------ 243
OsHKT1;1-V6   361 AAGTAACCCTTTGGTAGTTCAGCTCGTTTACTTTGTGATAATCTCATTTGCTGGTTTCCT 420
OsHKT1;1-V7   283 ------------------------------------------------------------ 283
OsHKT1;1-V8   244 ------------------------------------------------------------ 244

OsHKT1;1-FL   352 TGCTCTGAAGAATCTCAAGCCCCAGGGTAAGCCAGGTCCAAAGGATTTGGACCTGTTGTT 411
OsHKT1;1-V1   246 ---------GAATCTCAAGCCCCAGGGTAAGCCAGGTCCAAAGGATTTGGACCTGTTGTT 296
OsHKT1;1-V2   249 ----------AATCTCAAGCCCCAGGGTAAGCCAGGTCCAAAGGATTTGGACCTGTTGTT 298
OsHKT1;1-V3   352 TGCTCTGAAGAATCTCAAGCCCCAGGGTAAGCCGGGTCCAAAGGATTTGGACCTGTTGTT 411
OsHKT1;1-V4   349 TGCTCTGAAGAATCTCAAGCCCCAGGGTAAGCCAGGTCCAAAGGATTTGGACCTGTTGTT 408
OsHKT1;1-V5   244 ---------GAATCTCAAGCCCCAGGGTAAGCCAGGTCCAAAGGATTTGGACCTGTTGTT 294
OsHKT1;1-V6   421 TGCTCTGAAGAATCTCAAGCCCCAGGGTAAGCCAGGTCCAAAGGATTTGGACCTGTTGTT 480
OsHKT1;1-V7   284 ---------GAATCTCAAGCCCCAGGGTAAGCCAGGTCCAAAGGATTTGGACCTGTTGTT 334
OsHKT1;1-V8   245 ---------GAATCTCAAGCCCCAGGGTAAGCCAGGTCCAAAGGATTTGGACCTGTTGTT 295

OsHKT1;1-FL   412 CACCTCTGTGTCTACACTTACTGTCTCGAGCATGGCAACAGTAGAGATGGAAGACTTATC 471
OsHKT1;1-V1   297 CACCTCTGTGTCTACACTTACTGTCTCGAGCATGGCAACAGTAGAGATGGAAGACTTATC 356
OsHKT1;1-V2   299 CACCTCTGTGTCTACACTTACTGTCTCGAGCATGGCAACAGTAGAGATGGAAGACTTATC 358
OsHKT1;1-V3   412 CACCTCTGTGTCTACACTTACTGTCTCGAGCATGGCAACAGTAGAGATGGAAGACTTATC 471
OsHKT1;1-V4   409 CACCTCTGTGTCTACACTTACTGTCTCGAGCATGGCAACAGTAGAGATGGAAGACTTATC 468
OsHKT1;1-V5   295 CACCTCTGTGTCTACACTTACTGTCTCGAGCATGGCAACAGTAGAGATGGAAGACTTATC 354
OsHKT1;1-V6   481 CACCTCTGTGTCTACACTTACTGTCTCGAGCATGGCAACAGTAGAGATGGAAGACTTATC 540
OsHKT1;1-V7   335 CACCTCTGTGTCTACACTTACTGTCTCGAGCATGGCAACAGTAGAGATGGAAGACTTATC 394
OsHKT1;1-V8   296 CACCTCTGTGTCTACACTTACTGTCTCGAGCATGGCAACAGTAGAGATGGAAGACTTATC 355
                                                      M2
OsHKT1;1-FL   472 TGACAGGCAACTCTGGGTTCTGATCCTTCTGATGCTAATGGGAGGAGAGGTGTTCACATC 531
OsHKT1;1-V1   357 TGACAGGCAACTCTGGGTTCTGATCCTTCTGATGCTAATGGGAGGAGAGGTGTTCACATC 416
OsHKT1;1-V2   359 TGACAGGCAACTCTGGGTTCTGATCCTTCTGATGCTAATGGGAGGAGAGGTGTTCACATC 418
OsHKT1;1-V3   472 TGACAGGCAACTCTGGGTTCTGATCCTTCTGATGCTAATGGGAGGAGAGGTGTTCACATC 531
OsHKT1;1-V4   469 TGACAGGCAACTCTGGGTTCTGATCCTTCTGATGCTAATGGGAGGAGAGGTGTTCACATC 528
OsHKT1;1-V5   355 TGACAGGCAACTCTGGGTTCTGATCCTTCTGATGCTAATGGGAGGAGAGGTGTTCACATC 414
OsHKT1;1-V6   541 TGACAGGCAACTCTGGGTTCTGATCCTTCTGATGCTAATGGGAGGAGAGGTGTTCACATC 600
OsHKT1;1-V7   395 TGACAGGCAACTCTGGGTTCTGATCCTTCTGATGCTAATGGGAGGAGAGGTGTTCACATC 454
OsHKT1;1-V8   356 TGACAGGCAACTCTGGGTTCTGATCCTTCTGATGCTAATGGGAGGAGAGGTGTTCACATC 415

OsHKT1;1-FL   532 AATGCTAGGGCTCTACTTCAACAATGCCAATGCCAACAGAAATGAGAACAGCCAGAGAAG 591
OsHKT1;1-V1   417 AATGCTCGGGCTCTACTTCAACAATGCCAATGCCAACAGAAATGAGAACAGCCAGAGAAG 476
OsHKT1;1-V2   419 AATGCTAGGGCTCTACTTCAACAATGCCAATGCCAACAGAAATGAGAACAGCCAGAGAAG 478
OsHKT1;1-V3   532 AATGCTAGGGCTCTACTTCAACAATGCCAATGCCAACAGAAATGAGAACAGCCAGAGAAG 591
OsHKT1;1-V4   529 AATGCTAGGGCTCTACTTCAACAATGCCAATGCCAACAGAAATGAGAACAGCCAGAGAAG 588
OsHKT1;1-V5   415 AATGCTAGGGCTCTACTTCAACAATGCCAATGCCAACAGAAATGAGAACAGCCAGAGAAG 474
OsHKT1;1-V6   601 AATGCTAGGGCTCTACTTCAACA------------------------------------- 623
OsHKT1;1-V7   455 AATGCTAGGGCTCTACTTCAACAATGCCAATGCCAACAGAAATGAGAACAGCCAGAGAAG 514
OsHKT1;1-V8   416 AATGCTAGGGCTCTACTTCAACAATGCCAATGCCAACAGAAATGAGAACAGCCAGAGAAG 475

OsHKT1;1-FL   592 TTTACCTTCAATCAGCTTGGACATTGAATCCAACAGTCCTGCAAACAATGGGGATCACAA 651
OsHKT1;1-V1   477 TTTACCTTCAATCAGCTTGGACATTGAATCCAACAGTCCTGCAAACAATGGGGATCACAA 536
OsHKT1;1-V2   479 TTTACCTTCAATCAGCTTGGACATTGAATCCAACAGTCCTGCAAACAATGGGGATCACAA 538
OsHKT1;1-V3   592 TTTACCTTCAATCAGCTTGGACATTGAATCCAACAGTCCTGCAAACAATGGGGATCACAA 651
OsHKT1;1-V4   589 TTTACCTTCAATCAGCTTGGACATTGAATCCAACAGTCCTGCAAACAATGGGGATCACAA 648
OsHKT1;1-V5   475 TTTACCTTCAATCAGCTTGGACATTGAATCCAACAGTCCTGCAAACAATGGGGATCACAA 534
OsHKT1;1-V6   623 ------------------------------------------------------------ 623
OsHKT1;1-V7   515 TTTACCTTCAATCAGCTTGGACATTGAATCCAACAGTCCTGCAAACAATGGGGATCACAA 574
OsHKT1;1-V8   476 TTTACCTTCAATCAGCTTGGACATTGAATCCAACAGTCCTGCAAACAATGGGGATCACAA 535
                      Start codon
OsHKT1;1-FL   652 AATTACGGAATGTGGCCAATCAGAAGAAACTATGTCGCAAAACCAGGTACAGCAAAACAA 711
OsHKT1;1-V1   537 AATTACGGAATGTGGCCAATCAGAAGAAACTATGTCGCAAAACCAGGTACAGCAAAACAA 596
OsHKT1;1-V2   539 AATTACGGAATGTGGCCAATCAGAAGAAACTATGTCGCAAAAC----------------- 581
OsHKT1;1-V3   652 AATTACGGAATGTGGCCAATCAGAAGAAACTATGTCGCAAAACCAGATGCAAGAAA---- 707
OsHKT1;1-V4   649 AATTACGGAATGTGGCCAATCAGAAGAAACTATGTCGCAAAACCAGGTAC---------- 698
OsHKT1;1-V5   535 AATTACGGAATGTGGCCAATCAGAAGAAACTATGTCGCAAAACCAGGTAC --------- 580
OsHKT1;1-V6   623 ------------------------------------------------------------ 623
OsHKT1;1-V7   575 AATTACGGAATGTGGCCAATCAGAAGAAACTATGTCGCAAAACCAG-------------- 620
OsHKT1;1-V8   536 AATTACGGAATGTGGCCAATCAGAAGAAACTATGTC------------------------ 571
```

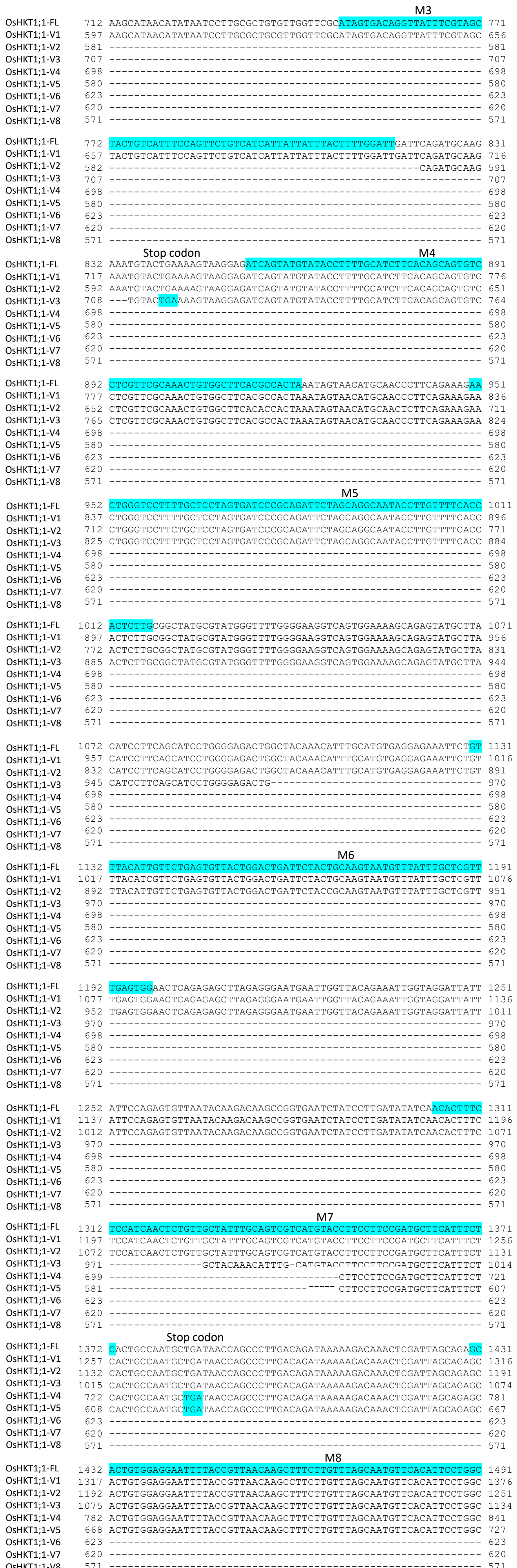
M3
M4
Stop codon
M5
M6
M7
Stop codon
M8
OsHKT1;1-FL
OsHKT1;1-V1
OsHKT1;1-V2
OsHKT1;1-V3
OsHKT1;1-V4
OsHKT1;1-V5
OsHKT1;1-V6
OsHKT1;1-V7
OsHKT1;1-V8

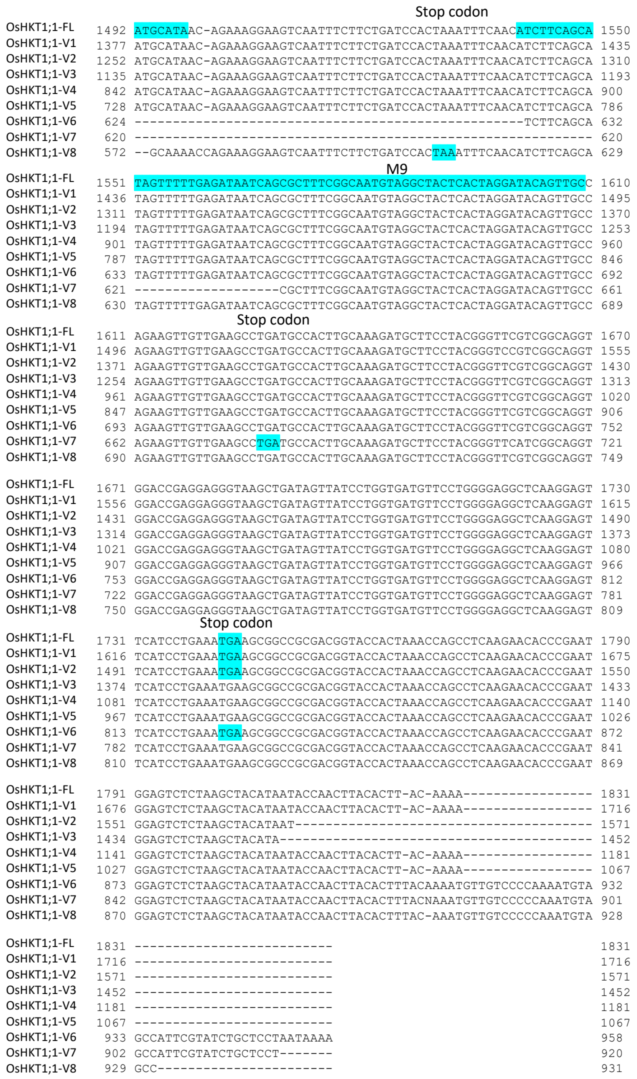

Supplementary Figure S3.

Nucleotide sequence alignment of OsHKT1;1-FL and its eight variant using GENETYX ver. 13. Presumed membrane-spanning regions of OsHKT1;1-FL, and positions of start and stop codon for the full-length and variants are indicated.

```
                                     
OsHKT1;1-FL    1 MHPPSLVLDTLKRIKLYIAMKLLLPNSEVLRIYWEKAQHLCGFLSMKLISRARCVASSVK 60
OsHKT1;1-V1    1 MHPPSLVLDTLKRIKLYIAMKLLLPNSEVLRIYWEKAQHLCGFLSMKLISRARCV----- 55
OsHKT1;1-V2    0 ------------------------------------------------------------ 0
OsHKT1;1-V3    1 MHPPSLVLDTLKRIKLYIAMKLLLPNSEVLRIYWEKAQHLCGFLSMKLISRARCVASSVK 60
OsHKT1;1-V4    1 MHPPSLVLDTLKRIKLYIAMKLLLPNSEVLRIYWEKAQHLCGFLSMKLISRARCVASSVK 60
OsHKT1;1-V5    1 MHPPSLVLDTLKRIKLYIAMKLLLPNSEVLRIYWEKAQHLCGFLSMKLISRARCV----- 55
OsHKT1;1-V6    1 MHPPSLVLDTLKRIKLYIAMKLLLPNSEVLRIYWEKAQHLCGFLSMKLISRARCVASSVK 60
OsHKT1;1-V7    1 MHPPSLVLDTLKRIKLYIAMKLLFPNSEVLRIYWEKAQHLCGFLSMKLISRARCV----- 55
OsHKT1;1-V8    1 MHPPSLVLDTLKRIKLYIAMKLLLPNSEVLRIYWEKAQHLCGFLSMKLISRARCV----- 55

                            M1
OsHKT1;1-FL   61 QSYSFLVCKSNPLVVQLVYFVIISFAGFLALKNLKPQGKPGPKDLDLLFTSVSTLTVSSM 120
OsHKT1;1-V1   56 --------------------------------NLKPQGKPGPKDLDLLFTSVSTLTVSSM 83
OsHKT1;1-V2    0 ------------------------------------------------------------ 0
OsHKT1;1-V3   61 QSYSFLVCKSNPLVVQLVYFVIISFAGFLALKNLKPQGKPGPKDLDLLFTSVSTLTVSSM 120
OsHKT1;1-V4   61 QSYSFLVCKSNPLVVQLVYFVIISFAGFLALKNLKPQGKPGPKDLDLLFTSVSTLTVSSM 120
OsHKT1;1-V5   56 --------------------------------NLKPQGKPGPKDLDLLFTSVSTLTVSSM 83
OsHKT1;1-V6   61 QSYSFLVCKSNPLVVQLVYFVIISFAGFLALKNLKPQGKPGPKDLDLLFTSVSTLTVSSM 120
OsHKT1;1-V7   56 --------------------------------NLKPQGKPGPKDLDLLFTSVSTLTVSSM 83
OsHKT1;1-V8   56 --------------------------------NLKPQGKPGPKDLDLLFTSVSTLTVSSM 83

                                M2
OsHKT1;1-FL  121 ATVEMEDLSDRQLWVLILLMLMGGEVFTSMLGLYFNNANANRNENSQRSLPSISLDIESN 180
OsHKT1;1-V1   84 ATVEMEDLSDRQLWVLILLMLMGGEVFTSMLGLYFNNANANRNENSQRSLPSISLDIESN 143
OsHKT1;1-V2    0 ------------------------------------------------------------ 0
OsHKT1;1-V3  121 ATVEMEDLSDRQLWVLILLMLMGGEVFTSMLGLYFNNANANRNENSQRSLPSISLDIESN 180
OsHKT1;1-V4  121 ATVEMEDLSDRQLWVLILLMLMGGEVFTSMLGLYFNNANANRNENSQRSLPSISLDIESN 180
OsHKT1;1-V5   84 ATVEMEDLSDRQLWVLILLMLMGGEVFTSMLGLYFNNANANRNENSQRSLPSISLDIESN 143
OsHKT1;1-V6  121 ATVEMEDLSDRQLWVLILLMLMGGEVFTSMLGLYFN------------------------ 156
OsHKT1;1-V7   84 ATVEMEDLSDRQLWVLILLMLMGGEVFTSMLGLYFNNANANRNENSQRSLPSISLDIESN 143
OsHKT1;1-V8   84 ATVEMEDLSDRQLWVLILLMLMGGEVFTSMLGLYFNNANANRNENSQRSLPSISLDIESN 143

                                                                 M3
OsHKT1;1-FL  181 SPANNGDHKITECGQSEETMSQNQVQQNKSITYNPCAVLVRIVTGYFVATVISSSVIIII 240
OsHKT1;1-V1  144 SPANNGDHKITECGQSEETMSQNQVQQNKSITYNPCAALVRIVTGYFVATVISSSVIIII 203
OsHKT1;1-V2    1 ------------------------------------------------------MWPIRR 6
OsHKT1;1-V3  181 SPANNGDHKITECGQSEETMSQNQMQEMY------------------------------- 209
OsHKT1;1-V4  181 SPANNGDHKITECGQSEETMSQNQVPSFRCFISHCQC----------------------- 217
OsHKT1;1-V5  144 SPANNGDHKITECGQSEETMSQNQVPSFRCFISHCQC----------------------- 180
OsHKT1;1-V6  156 ------------------------------------------------------------ 156
OsHKT1;1-V7  144 SPANNGDHKITECGQSEETMSQNQRFRQCRLLTRIQLPEVVEA----------------- 186
OsHKT1;1-V8  144 SPANNGDHKITECGQSEETMSQNQKGSQFLLIH--------------------------- 176

                                     M4                        M5
OsHKT1;1-FL  241 YFWIDSDARNVLKSKEISMYTFCIFTAVSSFANCGFTPLNSNMQPFRKNWVLLLLVIPQI 300
OsHKT1;1-V1  204 YFWIDSDARNVLKSKEISMYTFCIFTAVSSFANCGFTPLNSNMQPFRKNWVLLLLVIPQI 263
OsHKT1;1-V2    7 NYVAKPDARNVLKSKEISMYTFCIFTAVSSFANCGFTPLNSNMQLFRKNWVLLLLVIPHI 66
OsHKT1;1-V3  209 ------------------------------------------------------------ 209
OsHKT1;1-V4  217 ------------------------------------------------------------ 217
OsHKT1;1-V5  180 ------------------------------------------------------------ 180
OsHKT1;1-V6  156 ------------------------------------------------------------ 156
OsHKT1;1-V7  186 ------------------------------------------------------------ 186
OsHKT1;1-V8  176 ------------------------------------------------------------ 176

                                                               M6
OsHKT1;1-FL  301 LAGNTLFSPLLRLCVWVLGKVSGKAEYAYILQHPGETGYKHLHVRRNSVYIVLSVTGLIL 360
OsHKT1;1-V1  264 LAGNTLFSPLLRLCVWVLGKVSGKAEYAYILQHPGETGYKHLHVRRNSVYIVLSVTGLIL 323
OsHKT1;1-V2   67 LAGNTLFSPLLRLCVWVLGKVSGKAEYAYILQHPGETGYKHLHVRRNSVYIVLSVTGLIL 126
OsHKT1;1-V3  209 ------------------------------------------------------------ 209
OsHKT1;1-V4  217 ------------------------------------------------------------ 217
OsHKT1;1-V5  180 ------------------------------------------------------------ 180
OsHKT1;1-V6  156 ------------------------------------------------------------ 156
OsHKT1;1-V7  186 ------------------------------------------------------------ 186
OsHKT1;1-V8  176 ------------------------------------------------------------ 176

                                                               M7
OsHKT1;1-FL  361 LQVMFICSFEWNSESLEGMNWLQKLVGLLFQSVNTRQAGESILDISTLSPSTLLLFAVVM 420
OsHKT1;1-V1  324 LQVMFICSFEWNSESLEGMNWLQKLVGLLFQSVNTRQAGESILDISTLSPSTLLLFAVVM 383
OsHKT1;1-V2  127 PQVMFICSFEWNSESLEGMNWLQKLVGLLFQSVNTRQAGESILDISTLSPSTLLLFAVVM 186
OsHKT1;1-V3  209 ------------------------------------------------------------ 209
OsHKT1;1-V4  217 ------------------------------------------------------------ 217
OsHKT1;1-V5  180 ------------------------------------------------------------ 180
OsHKT1;1-V6  156 ------------------------------------------------------------ 156
OsHKT1;1-V7  186 ------------------------------------------------------------ 186
OsHKT1;1-V8  176 ------------------------------------------------------------ 176

                                                  M8
OsHKT1;1-FL  421 YLPSDASFLTANADNQPLTDKKTNSISRALWRNFTVNKLSCLAMFTFLACITERKSISSD 480
OsHKT1;1-V1  384 YLPSDASFLTANADNQPLTDKKTNSISRALWRNFTVNKPSCLAMFTFLACITERKSISSD 443
OsHKT1;1-V2  187 YLPSDASFLTANADNQPLTDKKTNSISRALWRNFTVNKLSCLAMFTFLACITERKSISSD 246
OsHKT1;1-V3  209 ------------------------------------------------------------ 209
OsHKT1;1-V4  217 ------------------------------------------------------------ 217
OsHKT1;1-V5  180 ------------------------------------------------------------ 180
OsHKT1;1-V6  156 ------------------------------------------------------------ 156
OsHKT1;1-V7  186 ------------------------------------------------------------ 186
OsHKT1;1-V8  176 ------------------------------------------------------------ 176

                              M9
OsHKT1;1-FL  481 PLNFNIFSIVFEIISAFGNVGYSLGYSCQKLLKPDATCKDASYGFVGRWTEEGKLIVILV 540
OsHKT1;1-V1  444 PLNFNIFSIVFEIISAFGNVGYSLGYSCQKLLKPDATCKDASYGSVGRWTEEGKLIVILV 503
OsHKT1;1-V2  247 PLNFNIFSIVFEIISAFGNVGYSLGYSCQKLLKPDATCKDASYGFVGRWTEEGKLIVILV 306
OsHKT1;1-V3  209 ------------------------------------------------------------ 209
OsHKT1;1-V4  217 ------------------------------------------------------------ 217
OsHKT1;1-V5  180 ------------------------------------------------------------ 180
OsHKT1;1-V6  157 -----IFSIVFEIISAFGNVGYSLGYSCQKLLKPDATCKDASYGFVGRWTEEGKLIVILV 211
OsHKT1;1-V7  186 ------------------------------------------------------------ 186
OsHKT1;1-V8  176 ------------------------------------------------------------ 176

OsHKT1;1-FL  541 MFLGRLKEFILK                                                 552
OsHKT1;1-V1  504 MFLGRLKEFILK                                                 515
OsHKT1;1-V2  307 MFLGRLKEFILK                                                 318
OsHKT1;1-V3  209 ------------                                                 209
OsHKT1;1-V4  217 ------------                                                 217
OsHKT1;1-V5  180 ------------                                                 180
OsHKT1;1-V6  212 MFLGRLKEFILK                                                 223
OsHKT1;1-V7  186 ------------                                                 186
OsHKT1;1-V8  176 ------------                                                 176
```

Supplementary Figure S4.

Protein sequence alignment of OsHKT1;1-FL and its eight variant using GENETYX ver. 13. Presumed membrane-spanning regions of OsHKT1;1-FL and its eight variants are indicated.

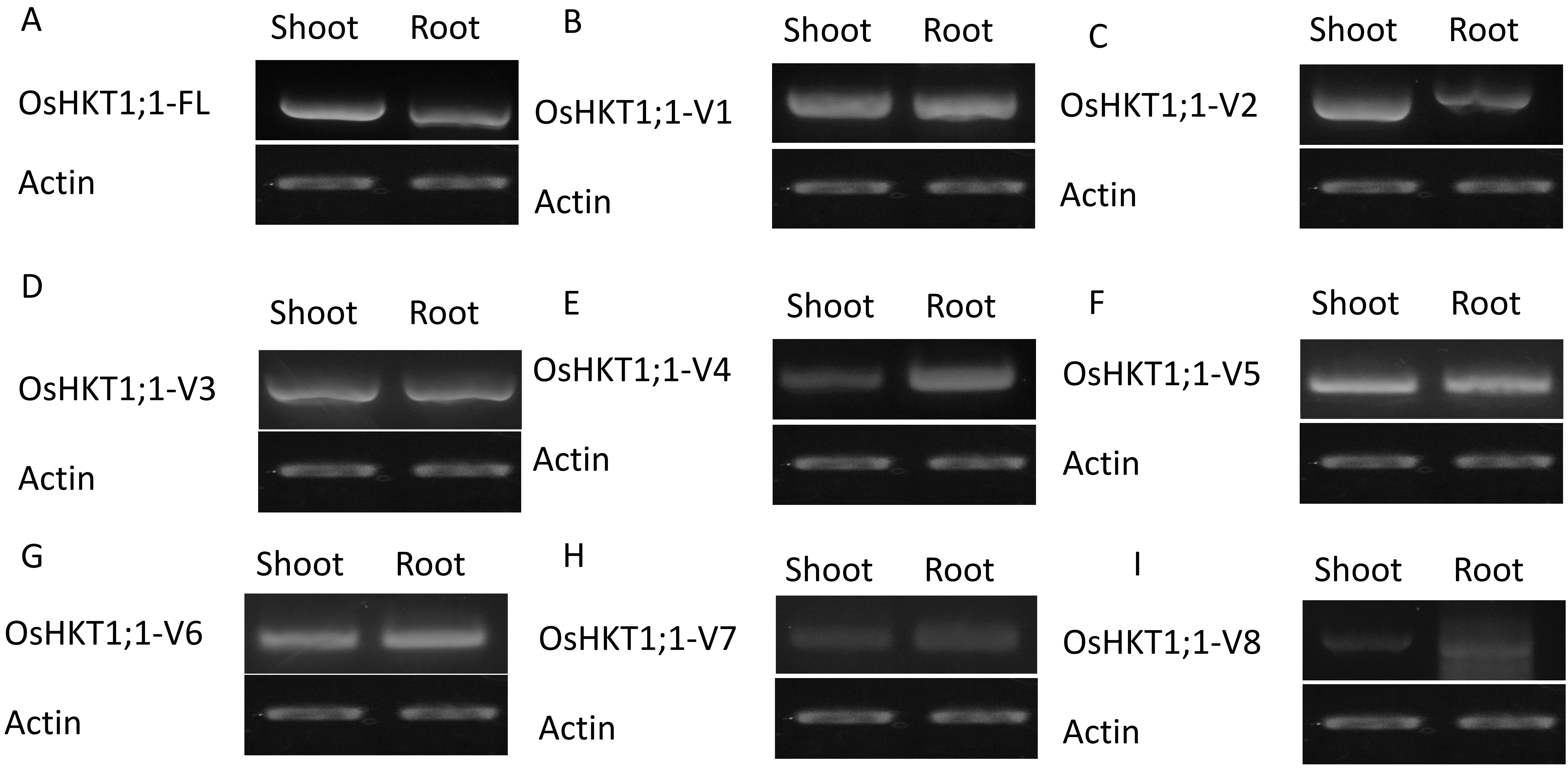

Supplementary Figure S5.
Expression analyzed with RT-PCR. OsHKT1;1-FL (A), OsHKT1;1-V1 (B), OsHKT1;1-V2 (C), OsHKT1;1-V3 (D), OsHKT1;1-V4 (E), OsHKT1;1-V5 (F), OsHKT1;1-V6 (G), OsHKT1;1-V7 (H), and OsHKT1;1-V8 (I) in “Pokkali” plants. Actin was used as an internal control.

```
OsHKT1;1-FL               1 MHPPSLVLDTLKRIKLYIAMKLLLPNSEVLRIYWEKAQHLCGFLSMKLISRARCVASSVK 60
OsHKT1;1-V1               1 MHPPSLVLDTLKRIKLYIAMKLLLPNSEVLRIYWEKAQHLCGFLSMKLISRARCV----- 55
OsHKT1;1-FL (ΔA56-K92)    1 MHPPSLVLDTLKRIKLYIAMKLLLPNSEVLRIYWEKAQHLCGFLSMKLISRARCV----- 55

OsHKT1;1-FL              61 QSYSFLVCKSNPLVVQLVYFVIISFAGFLALKNLKPQGKPGPKDLDLLFTSVSTLTVSSM 120
OsHKT1;1-V1              56 --------------------------------NLKPQGKPGPKDLDLLFTSVSTLTVSSM 83
OsHKT1;1-FL (ΔA56-K92)   56 --------------------------------NLKPQGKPGPKDLDLLFTSVSTLTVSSM 83

OsHKT1;1-FL             121 ATVEMEDLSDRQLWVLILLMLMGGEVFTSMLGLYFNNANANRNENSQRSLPSISLDIESN 180
OsHKT1;1-V1              84 ATVEMEDLSDRQLWVLILLMLMGGEVFTSMLGLYFNNANANRNENSQRSLPSISLDIESN 143
OsHKT1;1-FL (ΔA56-K92)   84 ATVEMEDLSDRQLWVLILLMLMGGEVFTSMLGLYFNNANANRNENSQRSLPSISLDIESN 143

OsHKT1;1-FL             181 SPANNGDHKITECGQSEETMSQNQVQQNKSITYNPCAVLVRIVTGYFVATVISSSVIIII 240
OsHKT1;1-V1             144 SPANNGDHKITECGQSEETMSQNQVQQNKSITYNPCAALVRIVTGYFVATVISSSVIIII 203
OsHKT1;1-FL (ΔA56-K92)  144 SPANNGDHKITECGQSEETMSQNQVQQNKSITYNPCAVLVRIVTGYFVATVISSSVIIII 203

OsHKT1;1-FL             241 YFWIDSDARNVLKSKEISMYTFCIFTAVSSFANCGFTPLNSNMQPFRKNWVLLLLVIPQI 300
OsHKT1;1-V1             204 YFWIDSDARNVLKSKEISMYTFCIFTAVSSFANCGFTPLNSNMQPFRKNWVLLLLVIPQI 263
OsHKT1;1-FL (ΔA56-K92)  204 YFWIDSDARNVLKSKEISMYTFCIFTAVSSFANCGFTPLNSNMQPFRKNWVLLLLVIPQI 263

OsHKT1;1-FL             301 LAGNTLFSPLLRLCVWVLGKVSGKAEYAYILQHPGETGYKHLHVRRNSVYIVLSVTGLIL 360
OsHKT1;1-V1             264 LAGNTLFSPLLRLCVWVLGKVSGKAEYAYILQHPGETGYKHLHVRRNSVYIVLSVTGLIL 323
OsHKT1;1-FL (ΔA56-K92)  264 LAGNTLFSPLLRLCVWVLGKVSGKAEYAYILQHPGETGYKHLHVRRNSVYIVLSVTGLIL 323

OsHKT1;1-FL             361 LQVMFICSFEWNSESLEGMNWLQKLVGLLFQSVNTRQAGESILDISTLSPSTLLLFAVVM 420
OsHKT1;1-V1             324 LQVMFICSFEWNSESLEGMNWLQKLVGLLFQSVNTRQAGESILDISTLSPSTLLLFAVVM 383
OsHKT1;1-FL (ΔA56-K92)  324 LQVMFICSFEWNSESLEGMNWLQKLVGLLFQSVNTRQAGESILDISTLSPSTLLLFAVVM 383

OsHKT1;1-FL             421 YLPSDASFLTANADNQPLTDKKTNSISRALWRNFTVNKLSCLAMFTFLACITERKSISSD 480
OsHKT1;1-V1             384 YLPSDASFLTANADNQPLTDKKTNSISRALWRNFTVNKPSCLAMFTFLACITERKSISSD 443
OsHKT1;1-FL (ΔA56-K92)  384 YLPSDASFLTANADNQPLTDKKTNSISRALWRNFTVNKLSCLAMFTFLACITERKSISSD 443

OsHKT1;1-FL             481 PLNFNIFSIVFEIISAFGNVGYSLGYSCQKLLKPDATCKDASYGFVGRWTEEGKLIVILV 540
OsHKT1;1-V1             444 PLNFNIFSIVFEIISAFGNVGYSLGYSCQKLLKPDATCKDASYGSVGRWTEEGKLIVILV 503
OsHKT1;1-FL (ΔA56-K92)  444 PLNFNIFSIVFEIISAFGNVGYSLGYSCQKLLKPDATCKDASYGFVGRWTEEGKLIVILV 503

OsHKT1;1-FL             541 MFLGRLKEFILK                                                 552
OsHKT1;1-V1             504 MFLGRLKEFILK                                                 515
OsHKT1;1-FL (ΔA56-K92)  504 MFLGRLKEFILK                                                 515
```

Supplement Figure S6.
Amino acid comparison among OsHKT1;1-FL, native OsHKT1;1-V1 and OsHKT1;1-FL (ΔA56-K92). GENETYX ver. 13 was used for exon-intron analysis.
